# Microbiota-activated CD103^+^ DCs stemming from microbiota adaptation specifically drive γδT17 proliferation and activation

**DOI:** 10.1186/s40168-017-0263-9

**Published:** 2017-04-24

**Authors:** Chris Fleming, Yihua Cai, Xuan Sun, Venkatakrishna R. Jala, Feng Xue, Samantha Morrissey, Yu-ling Wei, Yueh-hsiu Chien, Huang-ge Zhang, Bodduluri Haribabu, Jian Huang, Jun Yan

**Affiliations:** 1 Department of Microbiology and Immunology, University of Louisville School of Medicine, Louisville, KY USA; 2 Department of Medicine, James Graham Brown Cancer Center, University of Louisville School of Medicine, Louisville, KY USA; 3 Department of Microbiology and Immunology, Stanford University, Stanford, CA USA; 4 Department of Oncology, Zhejiang University the Second Affiliated Hospital, Hangzhou, China; 5 Tumor Immunobiology Program, James Graham Brown Cancer Center, University of Louisville School of Medicine, Louisville, KY USA

**Keywords:** Oral, Gut, Microbiome, Microbiota, Microbiota transfer, Germ-free, Fecal transplant, Host-microbiota interaction, IL-17, Gamma delta T cells, Dendritic cells

## Abstract

**Background:**

IL-17-producing γδT cells (γδT17) promote autoinflammatory diseases and cancers. Yet, γδT17 peripheral regulation has not been thoroughly explored especially in the context of microbiota-host interaction. The potent antigen-presenting CD103^+^ dendritic cell (DC) is a key immune player in close contact with both γδT17 cells and microbiota. This study presents a novel cellular network among microbiota, CD103^+^ DCs, and γδT17 cells.

**Methods:**

Immunophenotyping of IL-17r^−/−^ mice and IL-17r^−/−^ IRF8^−/−^ mice were performed by ex vivo immunostaining and flow cytometric analysis. We observed striking microbiome differences in the oral cavity and gut of IL-17r^−/−^ mice by sequencing 16S rRNA gene (v1–v3 region) and analyzed using QIIME 1.9.0 software platform. Principal coordinate analysis of unweighted UniFrac distance matrix showed differential clustering for WT and IL-17r^−/−^ mice.

**Results:**

We found drastic homeostatic expansion of γδT17 in all major tissues, most prominently in cervical lymph nodes (cLNs) with monoclonal expansion of Vγ6 γδT17 in IL-17r^−/−^ mice. Ki-67 staining and in vitro CFSE assays showed cellular proliferation due to cell-to-cell contact stimulation with microbiota-activated CD103^+^ DCs. A newly developed double knockout mice model for IL-17r and CD103^+^ DCs (IL-17r^−/−^IRF8^−/−^) showed a specific reduction in Vγ6 γδT17. Vγ6 γδT17 expansion is inhibited in germ-free mice and antibiotic-treated specific pathogen-free (SPF) mice. Microbiota transfer using cohousing of IL-17r^−/−^ mice with wildtype mice induces γδT17 expansion in the wildtype mice with increased activated CD103^+^ DCs in cLNs. However, microbiota transfer using fecal transplant through oral gavage to bypass the oral cavity showed no difference in colon or systemic γδT17 expansion.

**Conclusions:**

These findings reveal for the first time that γδT17 cells are regulated by microbiota dysbiosis through cell-to-cell contact with activated CD103^+^ DCs leading to drastic systemic, monoclonal expansion. Microbiota dysbiosis, as indicated by drastic bacterial population changes at the phylum and genus levels especially in the oral cavity, was discovered in mice lacking IL-17r. This network could be very important in regulating both microbiota and immune players. This critical regulatory pathway for γδT17 could play a major role in IL-17-driven inflammatory diseases and needs further investigation to determine specific targets for future therapeutic intervention.

**Electronic supplementary material:**

The online version of this article (doi:10.1186/s40168-017-0263-9) contains supplementary material, which is available to authorized users.

## Background

The IL-17 cytokine family has been extensively investigated in recent years and compelling evidence shows IL-17 plays a critical role in many diseases such as psoriasis and arthritis, as well as different types of cancer including colon, breast, ovarian, liver, and lung cancer [1–7]. In health, IL-17 contributes to the maintenance of epithelial integrity at the host-environment interface such as the lungs, skin, and intestine [8–10]. Innate production of IL-17 at the epithelial surface under homeostatic conditions is primarily from γδ T cells (γδT17) [11]. In-depth developmental and trafficking analyses of γδT17 cells suggest that the Vγ4^+^ population continually develop in the adolescent and adult thymus and constantly migrate to peripheral tissues. In contrast, Vγ6^+^ γδT17 stop developing in the thymus during embryogenesis and are maintained in the periphery as a self-replicating stem cell-like population [12, 13]. Development and trafficking aside, the regulation and maintenance of γδT17 cells in the periphery either through internal, self-propagating mechanisms or indirect, external players are still poorly understood.

Well-known γδT17 regulation primarily centers on direct and indirect innate processes including cytokine stimulation, pathogen-associated molecular pattern (PAMP) recognition, and complement receptor activation. γδT17 cells proliferate in response to various cytokines such as IL-1β and IL-7 [13, 14]. γδT17 can use PAMP receptors such as TLR2 for the induction of IL-17 responses further supporting innate-like characteristics [15]. Sepsis induction of C5a secretion resulted in DC-γδT17 crosstalk resulting in IL-17 production through a cytokine-dependent mechanism involving IL-6 and TGF-β [16]. Collectively, these studies suggest that γδT17 cells can be proliferated and expanded upon cytokine stimulation. However, it is unknown whether γδT17 cells in the periphery require cell-to-cell contact with other immune cells for homeostasis as well as induction of IL-17 secretion in response to tissue damage or microbial invasion.

A recently discovered and ubiquitously located antigen-presenting cell (APC) located at the same epithelial surfaces as γδT17 cells are CD103^+^ dendritic cells (DCs). This subset of DCs is a specialized yet heterogeneous population known to be potent inducers of CD8 T cell responses in most lymphoid tissues [17, 18]. CD8α^+^CD103^+^ DCs in the small intestine also induce Th1 response [19]. However, they are important inducers of FoxP3^+^ Tregs in the gut for oral tolerance [20, 21]. CD103^+^ DCs can take up antigen (Ag) from the environment in order to transport it to lymphoid tissues for Ag presentation [22, 23]. DC populations that cohabitate with γδT17 cells could be primary sources of regulation according to changing environmental cues and Ag sampling. During homeostasis, CD103^+^ DC endogenous activation of nearby γδT17 cells could be a major component of how our immune system maintains appropriate and healthy levels of microbiota at epithelial surfaces.

In the current study, we investigated how γδT17 cells are regulated during homeostasis especially in the absence of IL-17r signaling to and from the environment. We found that host-microbiota interactions both maintain γδT17 cells and induce their proliferation. In antibiotic-induced microbiota-depleted (AIMD) mice and germ-free (GF) mice, we observed a major collapse of the γδT17 population and the quality of their IL-17 responses, specifically in the Vγ6 γδT17 cells. Interestingly, microbiota appears to have extraordinary influence on γδT17 regulation in the cervical lymph nodes (cLN). We introduce a new cell-to-cell contact-dependent regulatory pathway between microbiota-activated CD103^+^ DCs and γδT17 cells. Clinically, this new regulatory network could play an important role in numerous disease models that may lead to novel and effective immunotherapies for a constantly expanding list of IL-17-driven inflammatory diseases dominated by the presence of γδT17 cells.

## Results

### γδT17 cell proliferation is peripherally regulated by the presence of IL-17r signaling

**Fig. 1:**
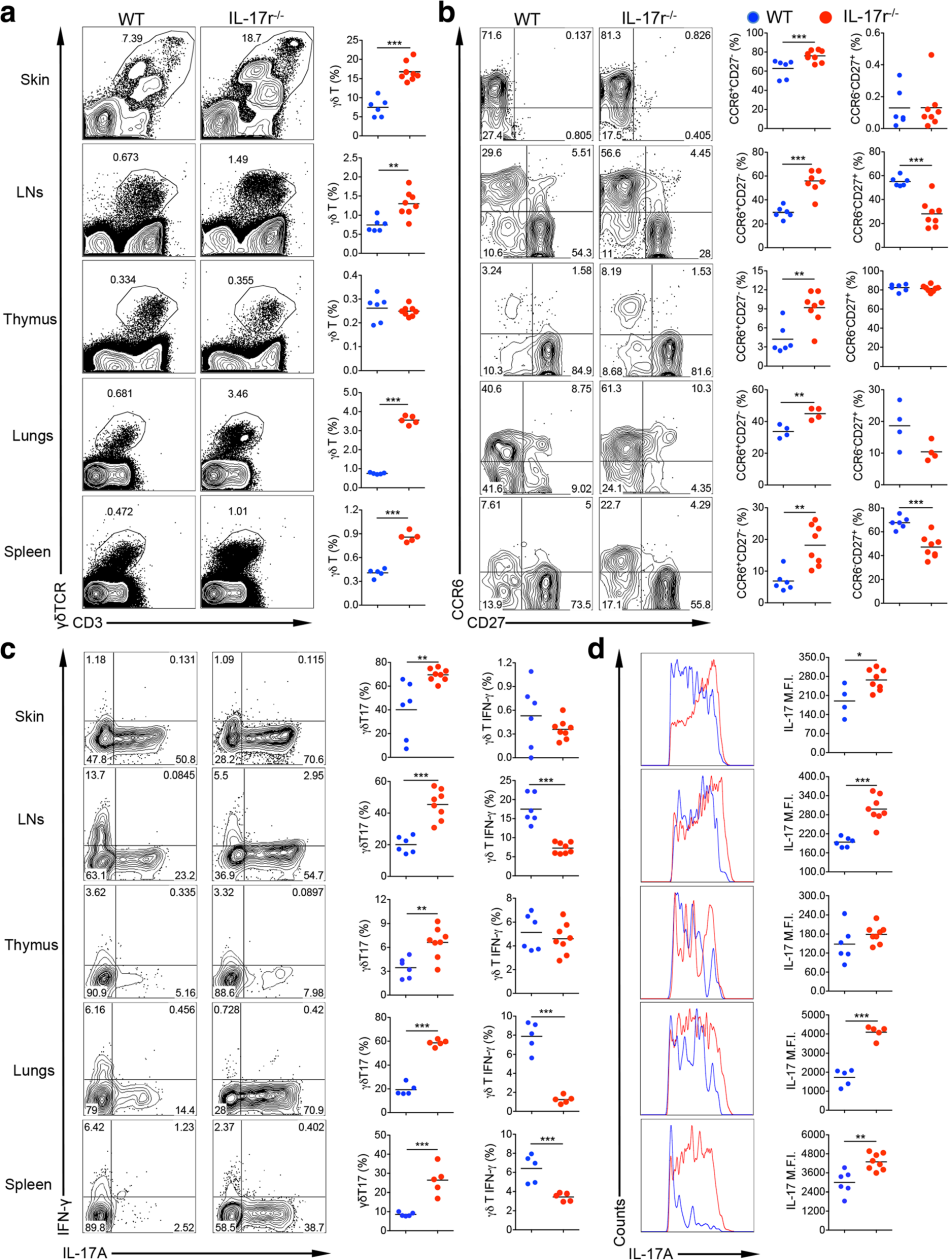
γδT17 cell expansion in mice deficient in IL-17 receptor. Flow cytometry studies staining homogenous tissue samples from different organs from IL-17r^−/−^ mice and WT mice of similar age and sex from the same facility. **a** Gating from the total live lymphocyte population then gated on CD3 versus γδTCR for total γδ T cell percentage in each tissue. *Plots* representative of seven to eight experiments. **p < 0.01, ***p < 0.001. **b** Gating from total γδ T cells then looking at CCR6 versus CD27 in each tissue to calculate CCR6^+^CD27^−^ (IL-17-capable γδ T cells) and CCR6^−^CD27^+^ (IFN-γ-capable γδ T cells). *Plots* representative of three experiments. **p < 0.01, ***p < 0.001. **c** Gated on total γδ T cells stimulated with PMA/Ionomycin for 5 h to calculate IL-17- or IFN-γ-producing γδ T cells. *Figures* representative of seven to eight experiments. **p < 0.01, ***p < 0.001. **d** Gated on IL-17-producing γδ T cells from IL-17r^−/−^ (*red*) and WT mice (*blue*) to compare mean fluorescence intensity (MFI) of IL-17 production level on a per cell basis. *Histograms* representative of seven to eight experiments. *p < 0.05, **p < 0.01, ***p < 0.001

We initially observed a drastic systemic expansion of total γδ T cells particularly γδT17 cells in IL-17r KO mice. To extensively dissect this phenotype, we performed ex vivo immunostaining on cell homogenate from the skin, lungs, body LNs, spleen, and thymus. We found an expansion of the total γδ T cell population in all tissues except the thymus (Fig. 1a). In addition, CCR6^+^CD27^−^ γδT cells, known for being capable of producing IL-17 [24, 25], were specifically increased (Fig. 1b). In contrast, CCR6^−^CD27^+^ γδT cells, which are capable of producing IFN-γ remained unchanged or significantly decreased (Fig. 1b). Consistent with CCR6 expression, in vitro γδT17 cells were increased in frequency (Fig. 1c) as well as IL-17 intensity on a per cell basis (MFI, Fig. 1d). Lack of total γδT cell expansion in the thymus suggests the expansion was regulated peripherally not centrally. Further support for peripheral expansion was the observation that γδ T cell expansion did not occur in the spleens and lungs till 2 days after birth (data not shown). Although the total percentage of CD4 T cells was not changed (Additional file 1: Figure S1A), CD4 T cells showed more polarization towards a Th17 phenotype (Additional file 1: Figure S1B, C). Similar γδT cell expansion in LNs was seen in IL-17A/F^−/−^ mice (Additional file 1: Figure S1D). This data shows that γδT17 cells are regulated by the presence of IL-17r signaling not only in the de novo synthesis of IL-17 [26] but also in the expansion of γδT17 cells in the periphery during homeostasis.

### Neck cervical LNs are specifically enlarged with monoclonal Vγ6 γδT17 cells in IL-17r^−/−^ mice

**Fig. 2:**
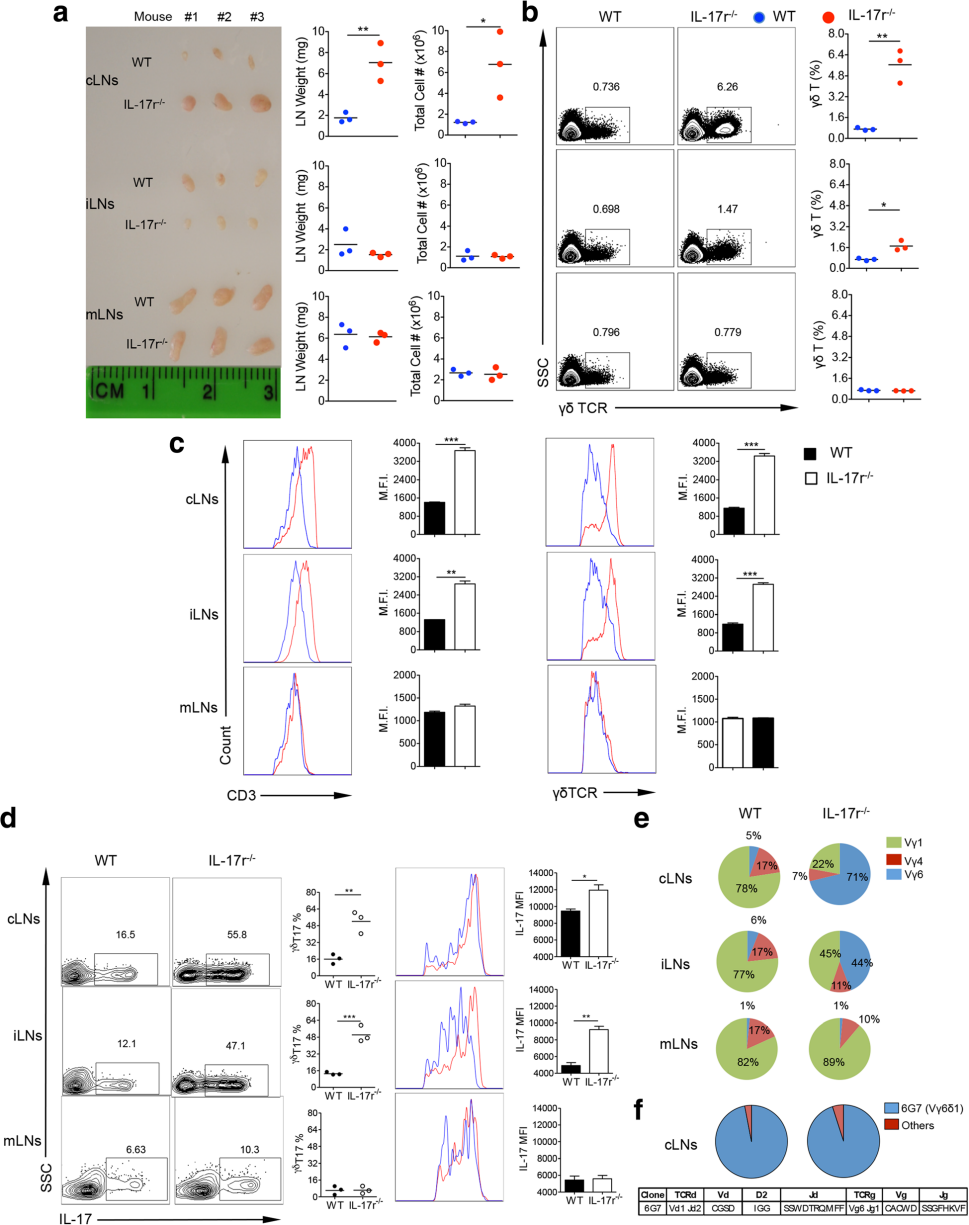
cLNs in the neck are specifically enlarged in IL-17r^−/−^ mice with expansion of a 6G7 clone of Vγ6 γδT17 cells. **a** *Photograph* showing the difference in size (scale shown in cm) of LNs between WT and IL-17r^−/−^ mice from three different locations with corresponding weight and total cell count from each LN. *Plots* representative of three different experiments. *p < 0.05, **p < 0.01. **b** Flow cytometry staining for γδ TCR from corresponding LN locations in (**a**) to calculate difference in total γδ T cell % between WT and IL-17r^−/−^ mice. Representative of four to five experiments. *p < 0.05, **p < 0.01. **c** Gated on total γδ T cells in the different LN locations and analyzed for CD3 and γδTCR surface expression intensity using MFI. Representative of three experiments. **p < 0.01, ***p < 0.001. **d** Gated on total γδ T cells after 5 h PMA/Ionomycin stimulation to analyze γδT17 frequencies. **p < 0.01, ***p < 0.001. **e** Frequencies of the three subsets of γδT cells Vγ6 (*blue*), Vγ1 (*green*), and Vγ4 (*red*). Pie charts representative of three different experiments. **f** Deep sequencing of individual clonal γδTCR expression on the Vγ6 γδ T cells sorted from cLNs of WT and IL-17r^−/−^ mice. Pie charts are representative of 192 clones from WT and 192 clones from IL-17r^−/−^

When harvesting tissues for ex vivo staining, we noticed in IL-17r-deficient mice that all tissues were of the same size and absolute cell number except the cervical LN (cLNs). cLN weight and total cell number were tripled but not inguinal (iLNs) and mesenteric (mLNs) LNs (Fig. 2a). In addition, a specific and drastic eightfold expansion of γδ T cells in the cLNs was revealed by flow cytometric analysis (Fig. 2b). Interestingly, the expanded γδ T cells had an increased expression of CD3 and γδTCR (Fig. 2c). Stimulating the total LN homogenate from the three different LN locations showed a higher frequency and MFI of IL-17 production in the cLNs and iLNs but not in the mLNs (Fig. 2d). It was previously reported that Vγ6δ1 γδ T cells have a higher expression of CD3 compared to other subsets [27], suggesting a change in the γδ T cell composition in the LN niche. By investigating the compositions of γδ T cell subsets in the three different LN locations, we found an extreme increase of Vγ6 γδ T cells in the cLNs and iLNs but not in the mLNs (Fig. 2e). The cLNs showed a drastic increase of Vγ6 γδ T cells from 5 to 71%. Utilizing a bar code enabled high-throughput ex vivo single-cell TCR sequence analysis to determine specific γδ TCR repertoire in the expanded cLN Vγ6 population, we found that approximately 95% of these γδ T cells in both wild type (WT) and IL-17r^−/−^ cLNs expressed a single pair of TCR sequences encoded by Vd1Dd2Jd1 and Vg6Jg1 with no N region diversity (Fig. 2f). Taken together, these data suggest that γδT17 homeostatic maintenance becomes dysregulated resulting in the specific expansion of a rare lymphatic Vγ6γδT17 subset in the absence of IL-17r signaling.

### γδT17 cells expand due to DC-dependent proliferation

**Fig. 3:**
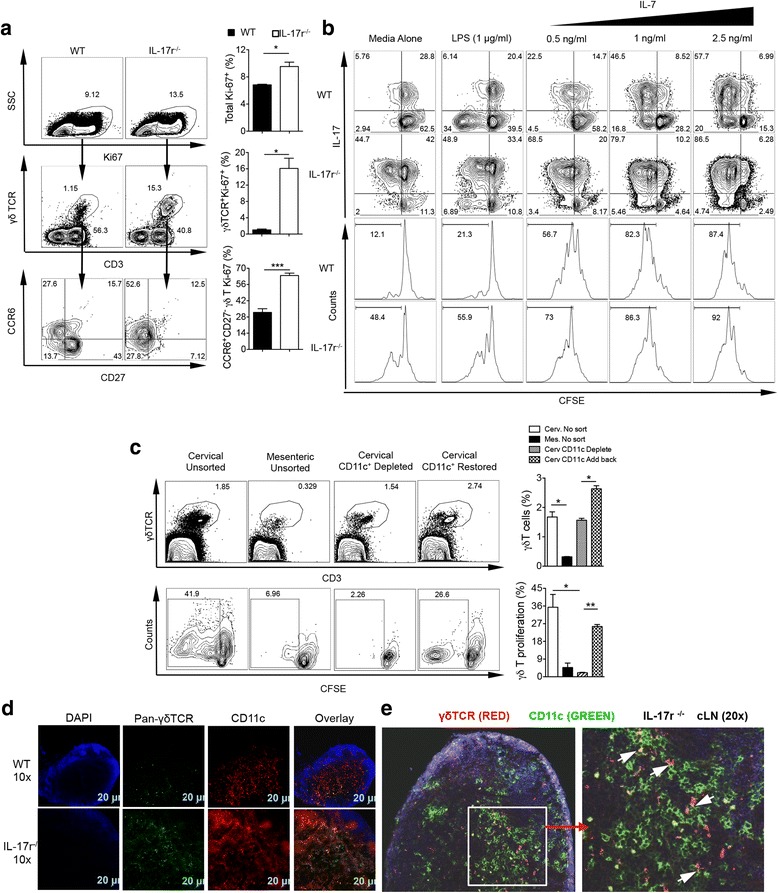
γδT17 cells expand due to DC-dependent proliferation. **a** Ex vivo staining of WT and IL-17r^−/−^ cLN cells for proliferation marker, Ki-67, to calculate total proliferating cells and contribution of γδ T and specifically CCR6^+^CD27^−^ γδ T to proliferation. Representative of two to three experiments. *p < 0.05, ***p < 0.001. **b** CFSE in vitro assay with no stimulation, LPS stimulation, and IL-7 titrated stimulation of total cLN cells from WT and IL-17r^−/−^ mice for 5 days then re-stimulated for 5 h with PMA/Ionomycin to examine proliferation of γδT17. Gated on total γδ T cells. *Dot plots* and *histograms* representative of five experiments. **c** Depletion of CD11c^+^ cells from IL-17r^−/−^ cLNs or adding back of CD11c^+^ cells to examine γδT cell proliferation after 5 days culture. Cells were gated on 7AAD^−^CD3^+^γδTCR^+^ cells. Representative of five experiments. *p < 0.05, **p < 0.01. **d** Immunohistochemistry staining of WT and IL-17r^−/−^ cLNs with pan-γδTCR (*green*), CD11c (*red*), and DAPI (*blue*) to visually show increase in γδ T cells and CD11c^+^DCs. Representative of cLNs from three different mice. Scale bar 20 μm. **e** High magnification (×20) of cLN image to show γδ T cells (*red*) and CD11c^+^DC (*green*) interaction in situ in IL-17r^−/−^ mice (*arrow*)

Different mechanisms may lead to a population’s expansion such as increased survival, less apoptosis, or proliferation. Ex vivo immunostaining for the proliferation protein Ki-67 confirmed specific and significant proliferation in CD27^−^CCR6^+^ γδ T cells (Fig. 3a). A previous study showed that IL-7 can specifically drive γδT17 expansion in the LNs [14]. Therefore, we examined whether γδT17 in IL-17r^−/−^ LNs responded more to IL-7 stimulation. Surprisingly, we observed that using whole cells from IL-17r^−/−^ mice after 5 days of culture led to spontaneous endogenous proliferation of γδ T cells without any stimulation, mainly γδT17 cells (Fig. 3b). Accounting for the endogenous proliferation, we did not see an advantage of γδT17 from IL-17r^−/−^ mice over WT when stimulated with LPS or IL-7. Endogenous proliferation was not observed in other tissues such as the lungs, spleen, or bone marrow (Additional file 1: Figure S2A).

This in vitro spontaneous proliferation system allowed us to specifically identify the mechanism of γδT17 proliferation. Endothelial cells have been shown to be the major producers of IL-7 [28]. However, by sorting out CD45^−^ cells from total cLN homogenate then culturing for 5 days, we observed no difference in the proliferation of γδT17 cells in vitro (Additional file 1: Figure S2B). We have previously shown that DCs are important inducers of γδT17 proliferation through the production of IL-1β/IL-23 [1, 3]. Therefore, we next examined whether γδ T proliferation in this system was dependent on DCs. Indeed, by depleting CD11c^+^ DCs, γδT17 cell proliferation was significantly reduced and when adding DCs back, we restored γδT17 proliferation (Fig. 3c). To further examine whether cLN from WT mice have similar effect, γδ T cells sorted from IL-17r KO mice were co-cultured with cLN from WT mice. Enhanced γδ T cell proliferation was not observed in the presence of WT cLN cells (Additional file 1: Figure S2C), suggesting that DCs from IL-17r KO mice may be activated. Next, we examined γδ T cell and DC interaction in situ in the IL-17r^−/−^ cLNs using immunofluorescence and confocal microscopy. We observed increased total γδ T cells as well as CD11c^+^ DCs validating our flow cytometry findings. At lower magnification, we did see increased colocalization between γδ T cells and DCs not seen in the WT control cLNs (Fig. 3d). At higher magnification in the IL-17r^−/−^ cLNs, close and intimate interactions between γδ T cells and DCs were readily seen (Fig. 3e). Thus, DCs from cLNs may directly interact and induce γδT17 proliferation and expansion.

### CD103^+^ DCs specifically induce Vγ6 γδT17 cell proliferation

**Fig. 4:**
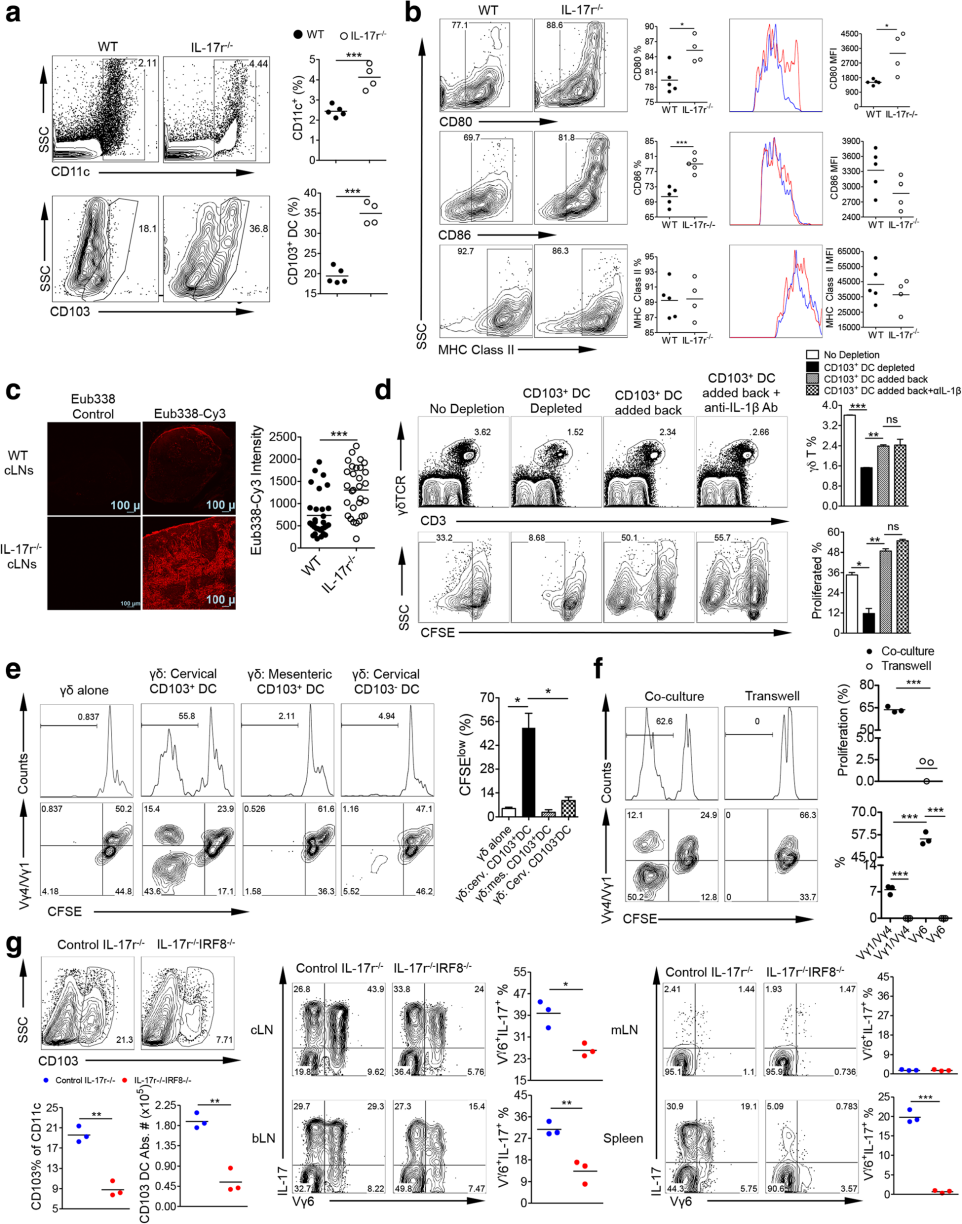
CD103^+^ DCs specifically induce Vγ6 γδT17 cell proliferation. **a** After ex vivo staining of cLN cells and gating from total live cells, CD11c^+^ cells were gated for calculating total DCs (*top*) then from this population CD103^+^ DCs were gated (*bottom*). Representative of two to three experiments. ***p < 0.001. **b** Gated on total CD103^+^ DCs from WT and IL-17r^−/−^ cLNs, staining of surface activation markers CD80, CD86, and MHC Class II was performed. Both percentages and MFI are shown. Representative of two to three experiments. *p < 0.05, ***p < 0.001. **c** 16S rRNA FISH hybridization on frozen tissue sections from WT and IL-17r^−/−^ cLNs. Probe Eub338-cy3 was used and TRIC signal intensity at random, yet the same locations for WT and IL-17r^−/−^ cLN was used to quantify the amount of bacterial RNA in LNs. Representative of three different cLNs from three mice. ***p < 0.001. **d** CFSE-labeled whole cLN cells were depleted of CD103^+^ DCs and cultured for 5 days. In conditions of whole cLN cells depleted with CD103^+^ DCs, sorted CD103^+^ DCs were added back in the presence or absence of anti-IL-1β neutralizing mAb (2 μg/ml). γδT cell proliferation was shown. Cells were gated on 7AAD^−^CD3^+^γδTCR^+^ cells. Representative of five experiments. *p < 0.05, **p < 0.01, ***p < 0.001. *n.s*. not significant. **e** Using MoFlo sorter, WT γδ T cells from the LNs and spleens were purified and CFSE labeled then co-cultured with three different IL-17r^−/−^ DC populations (CD103^+^ cLNs, CD103^+^ mLNs, and CD103^−^ cLNs) for 5 days. Representative histograms of γδT cell proliferation and summarized percentages of proliferated cells are shown (*upper panel*). Representative *dot plots* of proliferated γδT cells with Vγ4/Vγ1 staining are shown (*bottom panel*). Cells were gated on 7AAD^−^CD3^+^γδTCR^+^ cells. Representative of three experiments. *p < 0.05. **f** 96-well transwell plate was utilized to separate CD103^+^ DCs from cLNs of IL-17r^−/−^ mice from WT γδ T cells to determine if cell-to-cell contact is required. Representative histograms of γδT cell proliferation and summarized percentages of proliferated cells are shown (*upper panel*). Representative *dot plots* of proliferated γδT cells with Vγ4/Vγ1 staining and summarized data are shown (*bottom panel*). Cells were gated on 7AAD^−^CD3^+^γδTCR^+^ cells. Representative of three experiments. ***p < 0.001. **g** After immunostaining of cLNs, gated from total CD11c^+^ DCs then gated on CD103^+^ DC population to analyze total CD103^+^ DC % and absolute number. After PMA/ionomycin stimulation and intracellular staining, gated from total γδ T cells then gated on Vγ6^+^ and IL-17^+^ to calculate total Vγ6^+^ γδT17 %. Representative of two experiments. *p < 0.05, **p < 0.01, ***p < 0.001

DCs in LNs are composed of various subsets with different functions. We noticed that in cLNs, CD103^+^ DCs (CD11b^+^CD11c^hi^CD103^+^) were increased in frequency within total CD11c^+^ DCs (Fig. 4a). The increased CD103^+^ DCs were more activated with upregulated CD80 and CD86 expression levels but not MHC class II (Fig. 4b). To determine whether these DCs could be activated by bacterial Ags, we utilized 16S rRNA FISH hybridization and confocal microscopy to probe for bacterial 16S rRNA in the cLNs. The enlarged IL-17r^−/−^ cLNs showed a drastic increase in the presence of 16S rRNA compared to WT counterparts, suggesting that CD103^+^ DC activation could be due to bacterial product exposure (Fig. 4c).

To investigate whether CD103^+^CD11c^+^DCs drive γδT17 cell proliferation, we depleted this subset in vitro from the whole cLN using MoFlo sorter and cultured the cells without stimulation for 5 days. γδT17 cell proliferation was reduced by 75% after CD103^+^ CD11c^+^DCs depletion and restored by adding back CD103^+^CD11c^+^DCs to culture (Fig. 4d). In addition, IL-1β Ab blockade did not reduce γδT17 proliferation. To further determine whether γδT17 expansion can be induced by cLN CD103^+^ DCs, WT γδ T cells were co-cultured with different DC populations including CD103^+^ DCs either from cLNs or mLNs of IL-17r^−/−^ mice (Fig. 4e). Only CD103^+^ DCs from the cLNs were able to induce proliferation of the WT γδ T, and this CFSE^low^ population being negative for Vγ4 and Vγ1; thus, Vγ6^+^ is consistent with ex vivo cLN staining. Consistent with the IL-1β blockade results, γδT cell from IL-1r^−/−^ proliferate well when co-cultured with cLN CD103^+^ DCs (Additional file 1: Figure S2D), suggesting that γδT17 proliferation is not dependent on IL-1r signaling. In addition, neutralizing IL-23 did not significantly impact γδT17 proliferation (Additional file 1: Figure S2E). To examine whether CD103^+^ DCs drive γδT17 proliferation in vitro either through cell-to-cell contact or soluble factors, WT γδ T cells were co-cultured with CD103^+^ DCs from IL-17r^−/−^ mice in a transwell system. The co-cultured γδ T cells with the CD103^+^ DCs showed strong proliferation, predominately Vγ6. However, when separated from the CD103^+^ DCs, the WT γδ T cells did not proliferate (Fig. 4f), suggesting that a cell-to-cell interaction between DCs, specifically CD103^+^ DCs and γδT17 cells is required for the induction of their proliferation. To determine whether CD103^+^ DCs drive Vγ6 γδT17 expansion in vivo, IL-17r^−/−^ mice were backcrossed with IRF8^−/−^ mice, significant reduction in CD103^+^ DCs [29]. IL-17r^−/−^IRF8^−/−^ mice showed a reduction in total CD103^+^ DC % and absolute number which correlated with a large reduction in Vγ6 γδT17 in the cLNs, bLNs, and spleen but not the mLNs (Fig. 4g). This suggests that in vivo CD103^+^CD11c^+^DCs are important in the monoclonal expansion of Vγ6 γδT17 in IL-17r^−/−^ mice.

### Oral but not gut microbiota influences the expansion of γδT17 cells in the draining cLNs

Previous studies have shown an important role for microbiota in the expansion of γδT17 cells during various disease settings [3, 7, 30]. In the gut, the increase of SFB leads to increased frequency of Th17 cells [31]. The 16S rRNA gene sequencing data of fecal gut microbiota indeed showed that WT and IL-17r^−/−^ mice had distinct gut microbiota (Additional file 1: Figure S3A). In addition, IL-17r^−/−^ mice had increased SFB in the gut that correlated with increased Th17 cells in the lamina propria of the colon (Additional file 1: Figure S3B). However, we did not see a difference in γδ %, Vγ usage composition, or an increased γδT17 cells in the colon (Additional file 1: Figure S3B). To further examine the effect of gut microbiota on γδT17 cells, we transferred fecal samples from IL-17r^−/−^ mice to WT mice via gavage needle injection. However, we did not observe γδT17 expansion in the WT mice transplanted with IL-17r^−/−^ gut microbiota (Additional file 1: Figure S4).

**Fig. 5:**
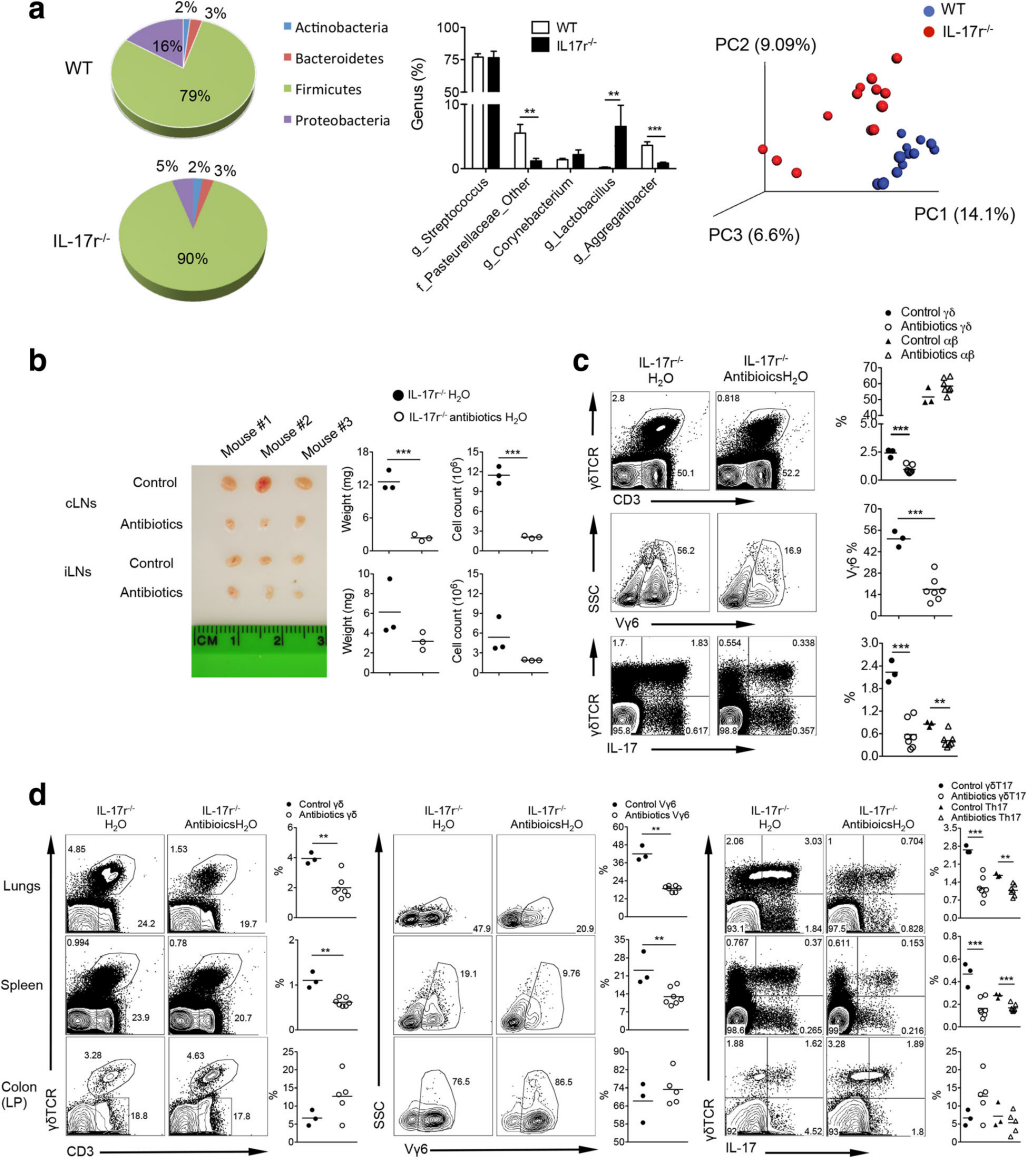
Oral microbiota influences the expansion of γδT17 cells in the draining cLNs. **a** The oral microbiota from WT and IL-17r^−/−^ mice was analyzed by sequencing 16S rRNA gene (v1–v3 region) and analyzed using QIIME 1.9.0 software platform. The abundant phylum and genus distribution between WT and IL-17r^−/−^ suggest alterations in the microbial composition. Principle coordinate analysis of unweighted UniFrac distance matrix showing the distinct clusters for WT (*blue*) and IL-17r^−/−^ (*red*) mice. ** *p* < 0.001; *** *p* < 0.0001. **b** cLNs (*upper*) and iLNs (*lower*) after oral antibiotics treatment for 6 weeks along with the corresponding weight of each node and the total cell number from that LNs. Representative of 2-3 experiments. ***p < 0.001. **c** Flow cytometry analysis showing percentages of γδ T cells, αβT cells, Vγ6 γδ T cells, and IL-17-producing γδ T and αβ T cells in the cLNs after 6 week oral antibiotics treatment from 1–2 days prior to birth to 6 weeks of age. Representative of two to three experiments. *p < 0.05, **p < 0.01, ***p < 0.001. **d** Flow cytometry analysis showing percentages of γδ T cells, Vγ6 γδ T cells, γδT17, and Th17 in the lungs, spleen, and colon (lamina propria) after 6-week oral antibiotics treatment from 1–2 days prior to birth to 6 weeks of age. Representative of two to three experiments. *p < 0.05, **p < 0.01, ***p < 0.001

Considering the drastic phenotype in the cLNs and the increase in migratory CD103^+^ DCs and bacterial products there, we investigated the effect of oral microbiota on γδT17 cell regulation. The overall oral microbiota composition at phylum and genus levels was significantly altered in IL-17r^−/−^ mice compared to WT (Fig. 5a). Specifically, *Lactobacillus* genus (Firmicutes phylum) were significantly increased in IL-17r^−/−^ mice compared to WT. In contrast, *Aggregatibacter* genus were significantly decreased in IL-17r^−/−^ mice. The statistical analysis of these distance matrices was performed using ANOSIM test with 999 permutations. The results demonstrated that both weighted and unweighted UniFrac distances were significantly different compared to each other indicating definitive differential microbiota composition between WT and IL-17r^−/−^ mice. We next utilized broad-spectrum antibiotic treatment (ampicillin, neomycin, vancomycin, and metronidazole) from prior to birth all the way to 6 weeks old to determine whether γδT17 expansion could be abrogated. Treatment with the oral antibiotic regimen prevented the increase in size and absolute cell number of the cLNs but did not affect the size and absolute number of cells in iLNs (Fig. 5b). γδ T cell %, Vγ6 %, and γδT17 % and absolute numbers were all decreased in the cLNs (Fig. 5c) as well as other peripheral tissues including the spleen and lungs but not in the colon (Fig. 5d). In addition to antibiotic depletion of microbiota, we utilized germ-free (GF) mice lacking microbiota which have reduced γδT17 population especially in the oral cLNs (Additional file 1: Figure S5A). Total IL-17-producing cells in the cLNs of GF mice were drastically lower compared to specific-pathogen-free (SPF) mice. In addition, Vγ6 γδT17 cells were significantly lower in GF mice compared to those in SPF mice. Immunostaining of DC populations showed a correlating decrease in total DCs and CD103^+^ DCs in the absence of microbiota (Additional file 1: Figure S5B). Together, these data suggest that microbiota in the oral cavity and not in the colon play an important role in the monoclonal proliferation of Vγ6 γδT17 cells leading to systemic expansion of γδT17 cells.

### Microbiota exchange induces γδT17 proliferation in the cLNs resulting in systemic expansion

**Fig. 6:**
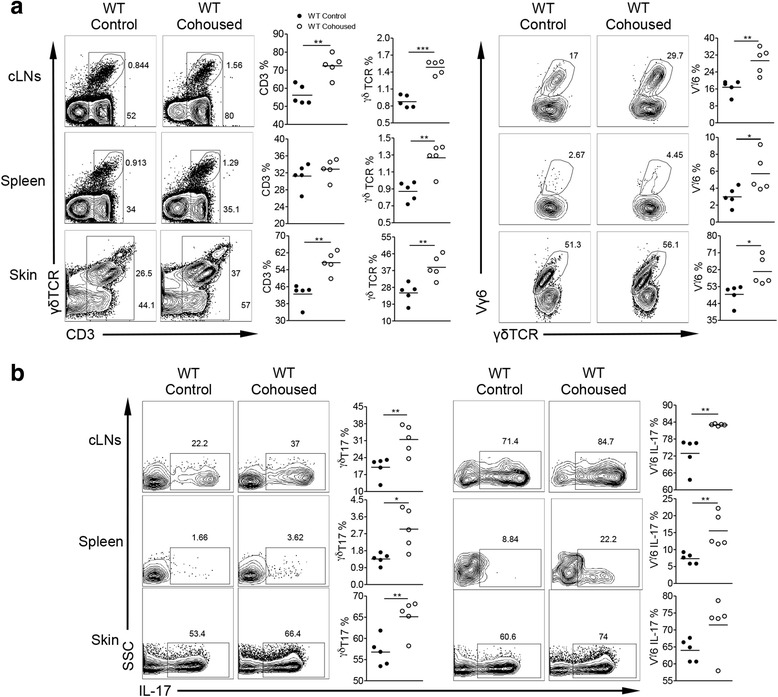
Co-housing of IL-17r^−/−^ mice with WT mice induces the proliferation of γδT17 cells in the cLNs and systemic expansion of γδT17 cells in WT mice. **a** Flow cytometry analysis of cell homogenate from different tissues examining the surface marker percentages of CD3, γδ T cells and Vγ6 γδ T cells between control and co-housed WT mice with IL-17r^−/−^ mice of the same age and sex. Representative *dot plots* and summarized data showing percentages of total CD3^+^ T cells, γδT cells, and Vγ6 T cells. *p < 0.05, **p < 0.01, ***p < 0.001. **b** Intracellular IL-17 expression between control and co-housed WT mice with IL-17r^−/−^ mice of the same age and sex. Representative *dot plots* and summarized data are shown. *p < 0.05, **p < 0.01

To examine the influence of microbiota on the peripheral regulation of γδT17 cells, without affecting the development of mucosal immunity, we co-housed animals together [32, 33]. WT mice were co-housed with IL-17r^−/−^ mice for 10 weeks after weaning. Co-housing resulted in increased CD3^+^ T cells, γδT cells, and Vγ6 T cells (Fig. 6a) in the WT mice. In addition, γδT17 cell proliferation and systemic expansion were seen in the co-housed WT mice (Fig. 6b). Conferral of γδT17 expansion by microbiota exchange was strongest in the cLNs and skin which also resulted in an increase in γδT17 cells in the spleen. This finding suggests that microbiota exchange can in fact induce the specific induction of Vγ6 γδT17 systemic expansion. Th17 cells were also increased in the skin and LNs however not in the spleen (Additional file 1: Figure S5C) again suggesting that the phenotype of systemic expansion is γδT17 specific. These data indicate that microbiota under homeostatic conditions do in fact modulate the proliferation and expansion of γδT17 cells.

## Discussion

In this study, we demonstrate that γδT17 cells are regulated through modulation of the microbiota with drastic, systemic changes in γδT17 frequency and IL-17-producing capabilities. The major regulatory pathway appears to be location and environment specific due to disproportionate expansion of γδT17 cells in the oral draining cLNs. We show that γδT17 cells are regulated in the cLNs through microbiota-activated CD103^+^ DCs in a cell-to-cell contact-dependent mechanism leading to monoclonal expansion of Vγ6 γδT17. Our in vivo double knockout model with deficiencies in IL-17r and IRF8 resulted in a drastic reduction in the CD103^+^CD11c^+^DC and Vγ6 γδT17 cell populations confirming that CD103^+^CD11c^+^DCs are important in regulating the proliferation and systemic expansion of Vγ6 γδT17 under dysbiotic conditions. This peripheral regulation network is initiated presumably at the mucosal surface and through CD103^+^CD11c^+^DC activation which leads to Vγ6 γδT17 proliferation in the draining cLNs and subsequently to systemic expansion in tissues such as the lungs, skin, and spleen but not in the colon or mLNs.

Previous studies have shown the ability of microbiota to influence the expansion of γδT17 cells [3, 7]. Here, we show that the expansion of γδT17 cells is transferred between mouse strains simply by co-housing WT and IL-17r^−/−^ mice. Microbiota differences between the IL-17r^−/−^ and WT mice can be explained by the idea of “microbiota adaptation.” In the absence of IL-17 signaling, as depicted here in IL-17r^−/−^ and IL-17r^−/−^IRF8^−/−^ mice, the microbiota and the immune system are trying to adapt and in essence reach a new equilibrium. Even if the microbiota do not have an IL-17r on their own membranes, they still respond to signals downstream of endogenous IL-17 signaling such as secretion of antimicrobial products HBD2 and calgranulin by keratinocytes [34]. Co-housing WT mice with IL-17r^−/−^ mice may force the WT mouse’s oral microbiota to adapt and reach a new equilibrium which in turn results in an adaptation and adjustment by the WT mouse’s immune system shown here as an expansion of γδT17 cells. Sequencing of oral and fecal samples showed distinct microbiota populations in both locations. Major differences were noted at the phylum and genus levels in the oral cavity compared with more moderate differences starting at the genus level in the gut. Even so, an important experiment to rule out gut microbiota contamination through consumption of fecal droppings was the fecal transfer experiment. The results show that gut microbiota as reported previously is important in Th17 induction in the intestine [31, 35, 36]. However, γδT17 cells are not affected by fecal transplant. Therefore, oral but not colonic microbiota is important in the peripheral regulation of γδT17. It is worth noting that further validating oral microbiota-driven γδT17 cell proliferation and expansion may require oral transfer of microbes directly in the future. It is also possible that skin microbiota may activate CD103^+^CD11c^+^DC in draining LNs thus partially contributing to such regulation. The prevalent behavior of murine grooming makes it exceptionally difficult to rule out the influence of skin microbiota on oral microbiota, and vice versa, when investigating host-microbiota regulatory effects.

With the treatment of IL-17r^−/−^ mice with broad-spectrum antibiotics through oral feeding, we completely abrogated γδT17 expansion in the periphery bringing the size and absolute cell number of IL-17r^−/−^ cLNs back to WT baseline levels, suggesting that oral microbiota is critical in regulating γδT17 proliferation and maintenance. In a more physiological setting, our GF mice studies show that Vγ6 γδT17 cells are at a minimal almost non-existent level in the absence of microbiota when compared to SPF mice even though total γδT cell frequency is not changed (data not shown). This suggests that microbiota plays a specific regulatory role in the peripheral maintenance of IL-17-producing Vγ6 γδT cells but not Vγ4 and/or Vγ1 γδT cells. We also observed a correlating drop in the CD103^+^CD11c^+^DC population in the cLNs in the absence of microbiota supporting our hypothesis that microbiota-regulated CD103^+^CD11c^+^DCs play a role in the maintenance and expansion of Vγ6 γδT17 cells.

DC cross talk with γδT17 cells using cytokines IL-1β and IL-23 to induce proliferation and IL-17 production during inflammatory settings has been well described [1, 3]. Our ex vivo depletion and co-culture studies were designed to determine what led to the proliferation of γδT17 cells in IL-17r^−/−^ mice during steady-state conditions. DCs, specifically the CD103^+^CD11c^+^DCs, in the cLNs of IL-17r^−/−^ can drive the proliferation of γδT17. This notion is further supported by the findings from IRF8^−/−^IL-17r^−/−^ mice where reduced CD103^+^CD11c^+^DCs in cLN lead to decreased γδT17 cells. Additionally, this supports our hypothesis that the observed proliferation and systemic expansion of γδT17 cells in the IL-17r^−/−^ mice is in fact location- and environment-dependent, perhaps due to “microbiota adaption” in the oral cavity. It is worth noting that deficiency of IRF8 in DCs results in reduced numbers of T cells in the small intestine [37]. CD103^+^CD11c^+^DCs are heterogeneous population. However, CD103^+^CD11c^+^DCs in cLN express CD11bMHCII (CD11c^+^CD11b^+^CD103^+^MHCII^hi^). This subset of DCs has been shown to exclusively instruct Th17 cells [38] Although CD103^+^CD11c^+^DC induction of γδT17 cell proliferation is independent of IL-1β or IL-23, a cell-to-cell contact is required for this process, at least in an ex vivo system. These are novel results considering how well established DC cytokine induction, specifically through IL-1β/IL-23, is for γδT17 activation and proliferation. Previous immunofluorescence microscopy studies showed direct cell-to-cell contact between DCs and Vγ4^−^Vγ1^−^ γδ T cells, and it was assumed that this interaction could potentially be important in regulation of γδ T cell function [39]. Our in vitro and in vivo findings both suggest a new regulatory network independent of soluble factors, dependent on APCs showing that γδT17 are not as socially inapt as once thought when compared to their siblings, the αβ T cells. Regulatory cross talk between γδT17 and CD103^+^ DCs either through cytokine or cell-to-cell interaction could be a major factor in how the immune system is able to respond and communicate with microbiota populations at the mucosal surface. Further investigation will be needed to determine through what mechanism-activated CD103^+^ DCs induce γδT17 proliferation.

One of the most confusing issues in γδT17 biology has been what events are needed for γδT17 activation and cytokine secretion [40]. While some γδ T cells responding to Ag stimulation in the periphery and undergoing Ag-specific differentiation into IL-17A-producing cells, such as phycoerythrin (PE)-specific γδ T cell response [41], other γδ T cells make IL-17 within hours after an immune challenge. The rapid IL-17 response does not show an apparent explicit TCR engagement but requires inflammatory cytokines such as IL-1/IL-23 for its induction [42]. These natural or innate-like γδT17 cells have a highly focused TCR repertoire regardless of their anatomical origin [43]. Wencker et al. reported previously that these γδ T cells require Ag recognition during thymic development which endows these cells to make IL-17 in response to cytokines in the periphery without engaging Ag [44]. Even though the expanded γδT17 cells in IL-17r^−/−^ mice have a highly invariant TCR repertoire, their expansion is cell-to-cell contact dependent via interaction with activated CD103^+^ DCs. It is still probable that they can recognize an array of different molecules depending on the molecular makeup and structure. Further investigation is needed into determining what Ags are important for the homeostatic upkeep of γδT17 cells as well as proliferative induction of γδT17 cells during disease.

Collectively, we propose a novel cellular pathway important in the regulation of host-microbiota interactions. γδT17 are important in the maintenance of epithelial barrier function and CD103^+^ migratory DCs are important in microbiota Ag sampling [30, 45–47]. Our findings connect the two processes and provide insights into how our body regulates this critical interface. Our results suggest that γδT17 and CD103^+^ DCs play a role of maintaining healthy microbiota populations at the epithelial surface in return being regulated themselves by the signals they receive from the microbiota. As the significance of γδT17 cells continue to grow in a constantly expanding list of diseases they are associated with, therapeutic solutions targeting γδT17 will become a necessity. Finding specific targets to modulate this critical interaction could be beneficial to the homeostatic maintenance of normal microbiota populations as well as to many patients suffering from systemic inflammatory diseases linked with microbiota dysbiosis.

## Conclusions

In this study, we demonstrate for the first time that γδT17 cells are regulated by microbiota dysbiosis through cell-to-cell contact with activated CD103^+^ DCs leading to drastic systemic, monoclonal expansion. Microbiota dysbiosis, as indicated by drastic bacterial population changes at the phylum and genus levels especially in the oral cavity, was discovered in mice lacking IL-17 receptor. This network could be very important in regulating both microbiota and immune players. This critical regulatory pathway for γδT17 could play a major role in IL-17-driven inflammatory diseases and needs further investigation to determine specific targets for future therapeutic intervention.

## Methods

### Mice

WT, IL-1r^−/−^ and IRF8^−/−^ mice on C57BL/6 background were originally purchased from Jackson Laboratory and homebred in our SPF murine facility. *Il17ra* ^−/−^ mice have been previously described [48]. GF mice and corresponding SPF mice were housed in the University of Chicago. All mice for comparison studies were homebred (6–8 weeks old) and housed in the University of Louisville’s SPF Clinical and Translational Research Mouse Facilities. All experimental mice were not ear tagged in this study except mice used for co-housing experiments. All animals were housed and treated in accordance with institutional guidelines and approved by the IACUC at the University of Louisville.

### Preparation of single-cell suspensions

Samples from different tissues were processed for single-cell suspensions. See Additional file 1: Supplemental Experimental Procedures for details.

### Ex vivo immunostaining and flow cytometry analysis

Cell staining procedures were detailed in the Additional file 1: Supplemental experimental procedures.

### Carboxyfluorescein succinimidyl ester (CFSE) labeling and co-culture assay

Single-cell homogenate from the LNs, spleen, lungs, and bone marrow were resuspended at a concentration of 1 × 10^7^ cells per ml and incubated with CFSE (1 μM) at 37 °C for 10 min. The reaction was then quenched by addition of ice-cold FBS. After CFSE labeling, 1 × 10^5^ γδ T cells were plated per well of a 96-well plate. 1 × 10^4^ DCs were added. After 5 days, the cells were harvested. In some experiments, neutralizing IL-1β or IL-23 mAbs were added in this culture system, or transwell plates were used. Cells were analyzed by flow cytometry.

### Co-housing and antibiotics in vivo studies

Co-housing protocols were described in Additional file 1: Supplemental experimental procedures.

### A single-cell gene sequencing

See Additional file 1: Supplemental experimental procedures for details.

### Oral and fecal microbiota sequencing

The detailed protocols for oral and fecal sample microbiota sequencing were described in Additional file 1: Supplemental experimental procedures.

### Statistical analysis

Results were exhibited as means ± SEM. Statistical analysis was performed using GraphPad Prism software. The statistical significance of differences between groups was determined by the Student’s *t* test. All data were analyzed using two-tailed tests unless otherwise specified, and a *P* value < 0.05 was considered statistically significant.

## Additional file

Additional file 1: Includes supplemental experimental procedures and five figures. (DOCX 2846 kb)

## Abbreviations

APC: Antigen-presenting cells
cLN: Cervical lymph nodes
DC: Dendritic cells
GF: Germ free
IL-17r: IL-17 receptor
iLN: Inguinal lymph nodes
IRF-8: Interferon regulatory factor 8
mLN: Mesenteric lymph nodes
PAMP: Pathogen-associated molecular pattern
SPF: Specific pathogen free
γδT17: IL-17-producing γδ T cells
