# Supplementary material for: Microbiota-activated CD103+ DCs stemming from microbiota adaptation specifically drive γδT17 proliferation and activation
Source: Microbiome. 2017 Apr 24;5:46. doi: 10.1186/s40168-017-0263-9 (PMC5404689; doi:10.1186/s40168-017-0263-9)
Supplement: Additional file 1: — Includes supplemental experimental procedures and five figures. (DOCX 2846 kb) [file 40168_2017_263_MOESM1_ESM.docx]

**Additional file 1**

1. Supplemental Experimental Procedures
2. Supplemental Figures (Total 5)

**Supplemental Procedures:**

**Preparation of single cell suspensions**

Mouse lungs were flushed with PBS, minced, digested with collagenase/hyaluronidase/DNAse digestion buffer for 45 min at 37 °C, and passed through a cell strainer to obtain a single cell suspension. Red blood cells (RBC) were lysed by ACK lysis buffer. Cells were then washed with complete RPMI. LNs and spleens were processed by meshing against a cell strainer using the plunger end of 5 ml syringe. RBCs were then lysed same as the lungs. Skin tissue was processed as described previously ^1^. Colon cecum and anal verge were harvested for cell preparations. Colon sections were thoroughly washed using repetitively PBS inversions in 50 mL tubes to remove all fecal material. After washes, colon samples were processed similar to the skin tissues.

***Ex vivo* immunostaining and flow cytometry analysis**

Mouse CD3, CD45, CD4, γδTCR, TCRVγ1, TCRVγ4, CD27, CD11c, CD11b, CD19, CD80, CD86, I-A/I-E, CD103, CD8, NK1.1., IFN-γ and IL-17A mAbs were obtained from Biolegend. Mouse CCR6 mAbs were obtained from R&Dsystem. Mouse Vγ6 TCR antibodies were kindly provided by Dr. Tigelaar (Clone 17D1, Yale University, New Haven, CT). For intracellular staining of cytokines, cells were stimulated with PMA and ionomycin in the presence of Golgiplug (BD-Bioscience) for 5 h and stained with different cell surface Abs and then fixed, permeabilized using Biolegend fixation/permeabilization buffers followed by staining intracellularly for IL-17 and IFN-γ. For RORγt intracellular staining Ebioscience FoxP3 fixation/permeabilization kit was utilized. The appropriate isotype control mAbs were also used.

**CD45 negative cell and DC depletion *ex vivo***

To deplete CD45 negative cells or DCs, single cell suspension from cLNs were stained for CD45, CD11c, CD103, and viability dye. CD45 negative live cells or CD45^+^CD11c^+^ DCs or CD45^+^CD11c^+^CD103^+^DCs were sorted using Beckman Coulter MoFlo sorter.

**Tissue sectioning and microscopy**

cLNs were frozen in OCT and stored at -80°C. Sections of approximately 7 µm were fixed with cold acetone for 15 min followed by 30 min of air dry. Slides were then blocked using 20% FBS in PBS for 1 h. Ab staining occurred overnight at 4°C in humidifier staining container. After 16 h staining, secondary Ab was added for 5 h at 4°C. For 16s RNA FISH hybridization, the slides were air dried and incubated with oligonucleotide probe Cy3-EUB338 (5 ng/µl). This probe (5′-GCT GCC TCC CGT AGG AGT-3′) is complementary to a region of the 16S rRNA, a highly conserved domain in *Bacteria.* The probe was labeled with Cy3 fluorophore at 5’ end (Integrated DNA Technologies, CA). The hybridization was carried out in the presence of 50 µl of 1x In situ hybridization buffer (Enzo) containing EUB338 probe (5 ng/µl). A large cover slip was placed on the slides and carefully pressed until the hybridization solution was evenly distributed over the respective section and incubated for 1 hr in humidified chamber at 46 ºC. The cover slip was carefully removed and the slides were rinsed with distilled water and incubated with DAPI containing anti-fade mounting media (BioMedia, CA). For immunofluorescent staining, cryosections were blocked with 5% BSA for 1 h and then stained with anti-mouse CD11c and pan γδ TCR mAbs (1:100 dilution) and DAPI overnight. Slides were washed with PBS. After air dry, Vecta mouse permanent mounting medium (Vector) was applied to slides with cover slips. The fluorescence images were captured using Nikon A1R confocal microscope with appropriate lasers.

**A single cell gene sequencing**

A single cell suspension from adult cLNs of WT and IL-17r^-/-^  mice were immunostained for CD3, pan δTCR, and Vγ6. A single CD3^+^γδ^+^Vγ6^+^ cell was sorted into 96-well plate containing 10 µl Qiagen One Step PCR 1x Buffer using Beckman Coulter MoFlo. Plates were spun down at 3000 rpm for 2 min then frozen at -20 °C immediately. RT-PCR for Vγ6 TCR gene sequence was performed at Stanford University using Barcode enabled high throughput single cell TCR determination described previously ^2^. For IL-17r^-/-^ mice, total 150 cells were sorted and 143 productive sequences were obtained while in WT mice total 147 cells were sorted and 140 productive sequences were obtained.

**Co-housing and antibiotics *in vivo* studies**

WT and IL-17r^-/-^  mice once reaching 4 weeks old were separated into either cohoused groups or WT alone and IL-17r^-/-^ alone control groups. The mice were cohoused for 10 weeks and then were euthanized. In the antibiotics protocol, a pregnant IL-17r^-/-^  female 1-2 days prior to delivering pups was given fresh antibiotics water described previously ^3^ consisting of Ampicillin (1 g/L), Vancomycin (500 mg/L), Neomycin Sulfate (1 g/L) [GoldBio] and Metronidazole (1 g/L) [Sigma]. The female was given antibiotics water till delivery and pups were maintained on antibiotics water till 6 weeks of age when the experiment reached the terminal timepoint.

**Oral and fecal microbiota DNA isolation and sequencing**

The v1-v3 regions of 16S rRNA gene was amplified using 27f (AGAGTTTGATCCTGGCTCAG) and 534r (ATTACCGCGGCTGCTGG) primers (1 μM). The primers were anchored adaptor (adopter A: 5’ CCATCTCATCCCTGCGTGTCTCCGACTCAG 3’ and adopter B: 5’ CCTATCCCCTGTGTGCCTTGGCAGTCTCAG 3’) and Multiplex Identifiers (MIDs; 10 bp long). The multiplexed amplicons were gel purified and sequenced using the 454 Jr. Sequencing platform. The 16S rRNA sequences were analyzed using QIIME (1.9.0) platform scripts ([www.qiime.org](http://www.qiime.org/)) ^4^. The sequences were rarified at randomly selected 2000 sequences/sample and downstream analysis was performed. The microbial classification was performed using Green Genes reference database (gg_13_8_otus) using QIIME tools ^4^. The sequences reference picked into Operational Taxonomic Units (OTUs) by clustering 97% sequence similarity (uclust) and classified at various taxonomic ranks (phylum, order, class, family, genus, and species). The beta diversity principle co-ordinate plots were generated using phylogenetic metrics of UniFrac distances. The Unifrac metric distances were used to calculate the significance between two groups using ANOSIM with 999 permutations. The oral and fecal microbiota datasets were deposited in the Biosample database with submission ID: SUB2183894 and SUB2183406, respectively.

**In vivo fecal transfer studies**

The recipient adult male mice were treated for 1 week with broad-spectrum anti-biotics. The cocktail of antibiotics were prepared in drinking water at following concentrations: Ampicillin (1 gm/l), Vancomycin (500 mg/l), Neomycin Sulfate (1 g/l) and Metronidazole (1 gm/l) as described previously ^3, 5, 6, 7^. The fecal pellets from individual mice (3-4 mice of each genotype) were collected freshly and pooled the pellets and weighed. The pellets were resuspended in transfer buffer, sterile filtered 0.05% cysteine HCl (Calbiochem/EMD Millipore, Billerica, MA) in Dulbecco’s Phosphate Buffered Saline (PBS). The final volume was adjusted to give 120 mg fecal sample per ml. The fecal pellets were mashed with sterile wooden tooth picks and vortexed for 1 min. The samples were centrifuged at 800 x g speed for 3 min. 100 µl of this supernatant per mouse was oral gavaged for each fecal transfer experiment. One hour prior to fecal transplant, mice were injected IP with 3 mg of cimetidine HCl and 0.02 mg of sincalide to reduce gastric acid levels and enhance reconstitution *^8^.* WT mice were transplanted with either IL-17r^-/-^ or WT fecal samples. We performed fecal transfers once per week for 3 wks followed by 4 wks of rest for gut microbiota reconstitution.

**References:**

1. Cai, Y. *et al.* Pivotal role of dermal IL-17-producing gammadelta T cells in skin inflammation. *Immunity* **35**, 596-610 (2011).

2. Wei, Y.L. *et al.* A Highly Focused Antigen Receptor Repertoire Characterizes gammadelta T Cells That are Poised to Make IL-17 Rapidly in Naive Animals. *Frontiers in immunology* **6**, 118 (2015).

3. Rakoff-Nahoum, S., Paglino, J., Eslami-Varzaneh, F., Edberg, S. & Medzhitov, R. Recognition of commensal microflora by toll-like receptors is required for intestinal homeostasis. *Cell* **118**, 229-241 (2004).

4. Caporaso, J.G. *et al.* QIIME allows analysis of high-throughput community sequencing data. *Nature methods* **7**, 335-336 (2010).

5. Garrett, W.S. *et al.* Communicable ulcerative colitis induced by T-bet deficiency in the innate immune system. *Cell* **131**, 33-45 (2007).

6. Mukherji, A., Kobiita, A., Ye, T. & Chambon, P. Homeostasis in intestinal epithelium is orchestrated by the circadian clock and microbiota cues transduced by TLRs. *Cell* **153**, 812-827 (2013).

7. Tsay, T.B., Yang, M.C., Chen, P.H., Hsu, C.M. & Chen, L.W. Gut flora enhance bacterial clearance in lung through toll-like receptors 4. *Journal of biomedical science* **18**, 68 (2011).

8. Iida, N. *et al.* Commensal bacteria control cancer response to therapy by modulating the tumor microenvironment. *Science* **342**, 967-970 (2013).

**Supplemental Figure 1**


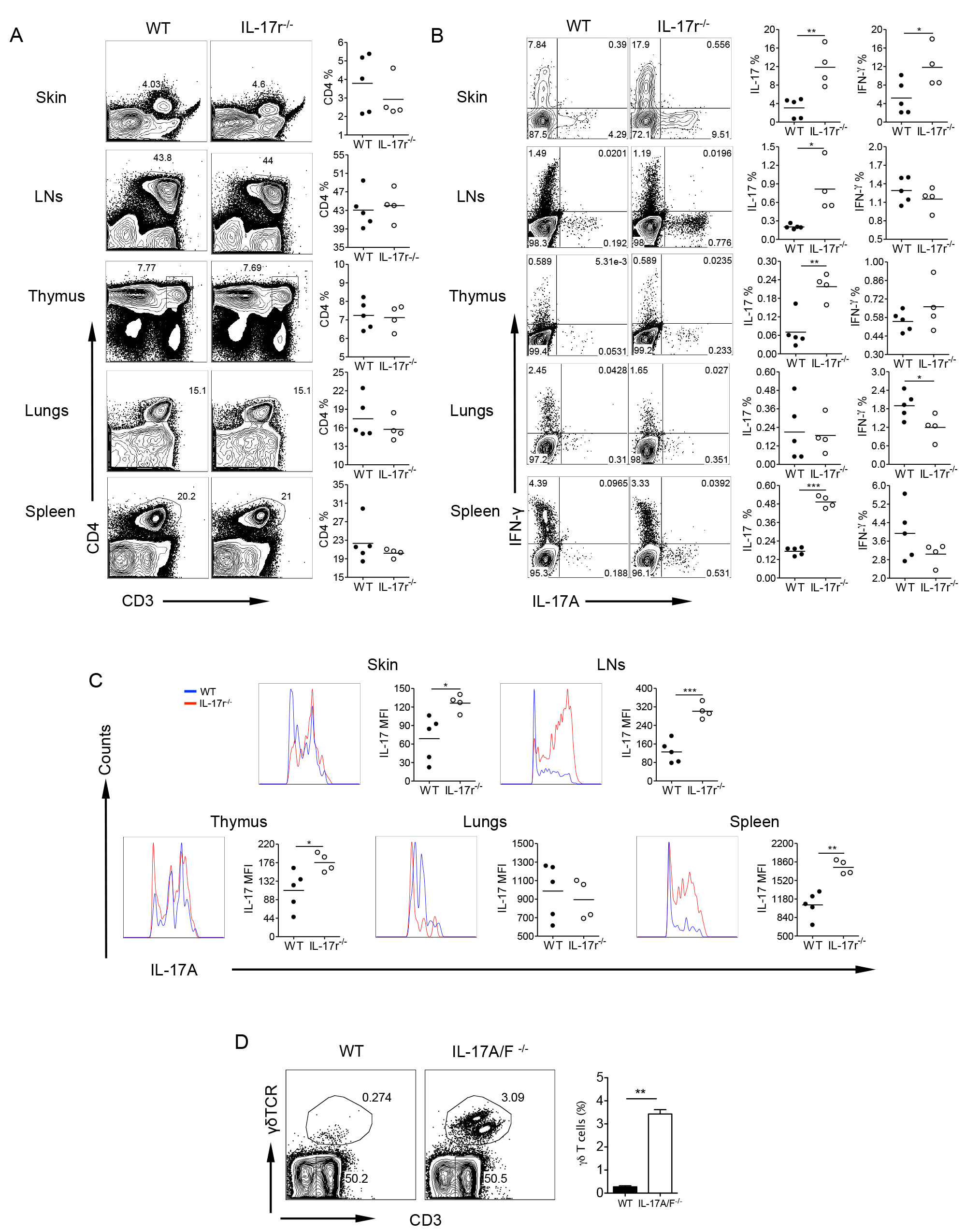


**Figure S1. Th17 polarization increased with IL-17 signaling deficiency**

Flow cytometry studies staining homogenous tissue samples from different organs from IL-17r^-/-^ and WT mice of similar age and sex from the same facility.

1. Gating from the total live lymphocyte population and then gated on CD3 versus CD4 for total CD4^+^ T cell percentage in each tissue. Plots representative of 7-8 experiments.
2. Gated on total CD4^+^ T cells stimulated with PMA/Ionomycin for 5 h to calculate IL-17- or IFN-γ-producing cells. Figures representative of 7-8 experiments. *p<0.05, **p<0.01, ***p<0.001
3. Gated on total IL-17-producing CD4^+^ T cells from WT (blue) and IL-17r^-/-^ (red) mice and looking at mean fluorescence intensity (MFI) to calculate IL-17 production on a per cell basis in different tissues. Histograms representative of 7-8 experiments. *p<0.05, **p<0.01, ***p<0.001
4. Cervical lymph nodes from WT and IL-17A/F^-/-^ mice (n=2) were stained with CD3 and γδTCR. Cells were gated on the total live lymphocyte population. Representative plots and summarized percentages of γδ T cells are shown. **p<0.01

**Supplemental Figure 2**


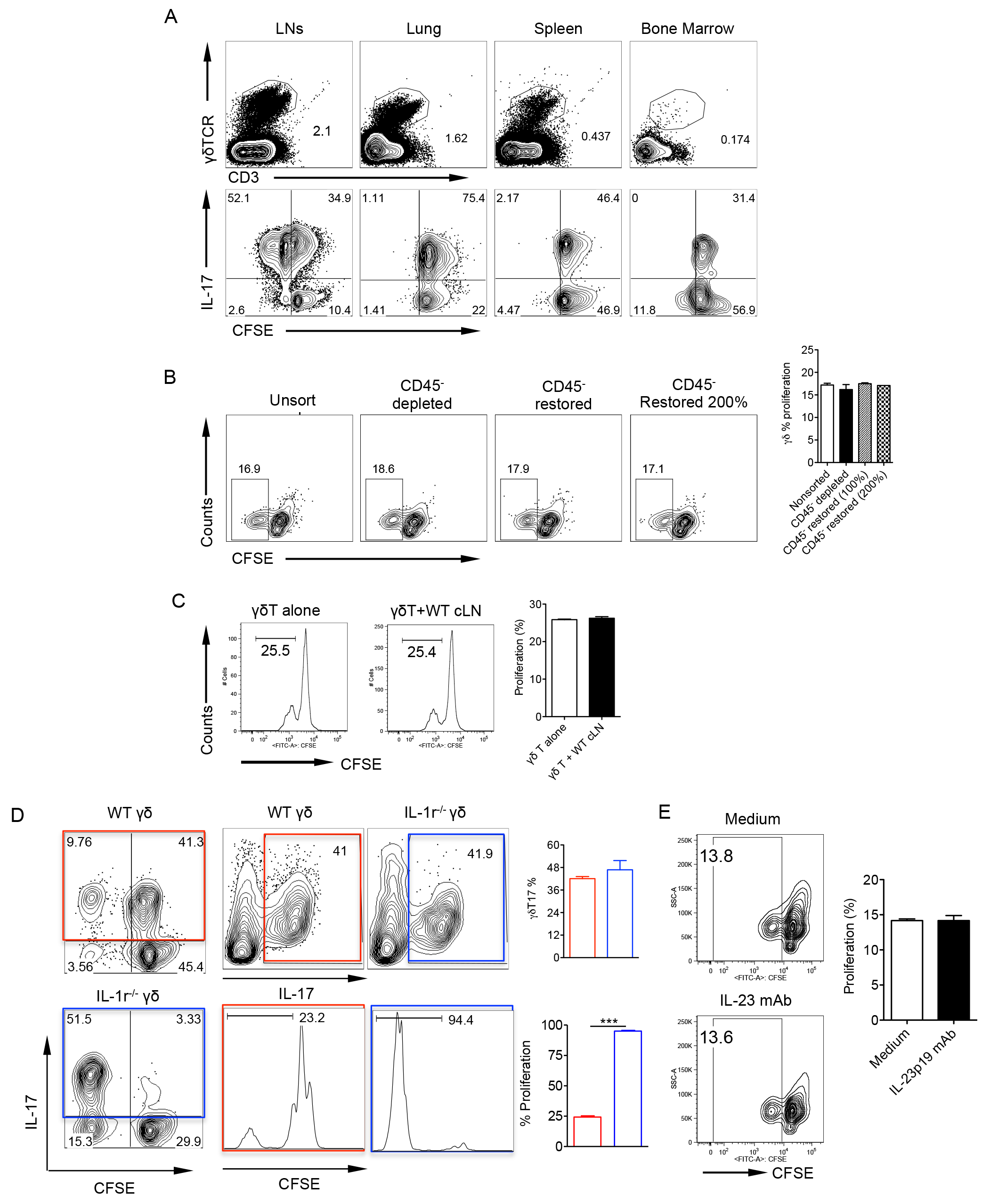


**Figure S2. LN specific endogenous proliferation of γδT17 cells is independent of CD45^-^ cells and IL-1r expression on γδT cells.**

(A) Gated on total live lymphocyte population in different tissues then from that gate analyzed the γδT17 proliferation using IL-17 vs CFSE gating. Representative of 2-3 experiments.

(B) Depleted CD45^-^ cells from IL-17r^-/-^ LNs using Facs Aria to determine CD45^-^ cell contribution to γδT17 cell endogenous proliferation after 5 days culture. Representative of 3 experiments.

(C) γδ T cells sorted from IL-17r^-/-^ mice were CSFE labeled and then co-culture with or without cervical lymph node cells from WT mice at 1:1 ratio for 5 days. Γδ T cell proliferation was determined by flow cytometry.

(D) CD103^+^ DCs were co-cultured with WT and IL-1r^-/-^ γδ T cells (1:5) to determine the role of γδT IL-1r expression in the proliferation of γδT17 (left panel). Gated on total γδT population and then showed total IL-17^+^ cells after 5 days co-culture (right, upper panel). Gated on total γδT17 and then gated for CFSE to analyze the % of proliferated γδT17 from co-cultures (right, bottom panel). Representative of 3 experiments. **p<0.01

(E) Cervical lymph node cells were labeled with CSFE and then cultured in the presence or absence of neutralizing IL-23p19 mAb (12.5 μg/ml) for 5 days. Cells were gated on CD3^+^ γδTCR^+^ T cells. Representative histograms and summarized data (n=3) are shown.

**Supplemental Figure 3**


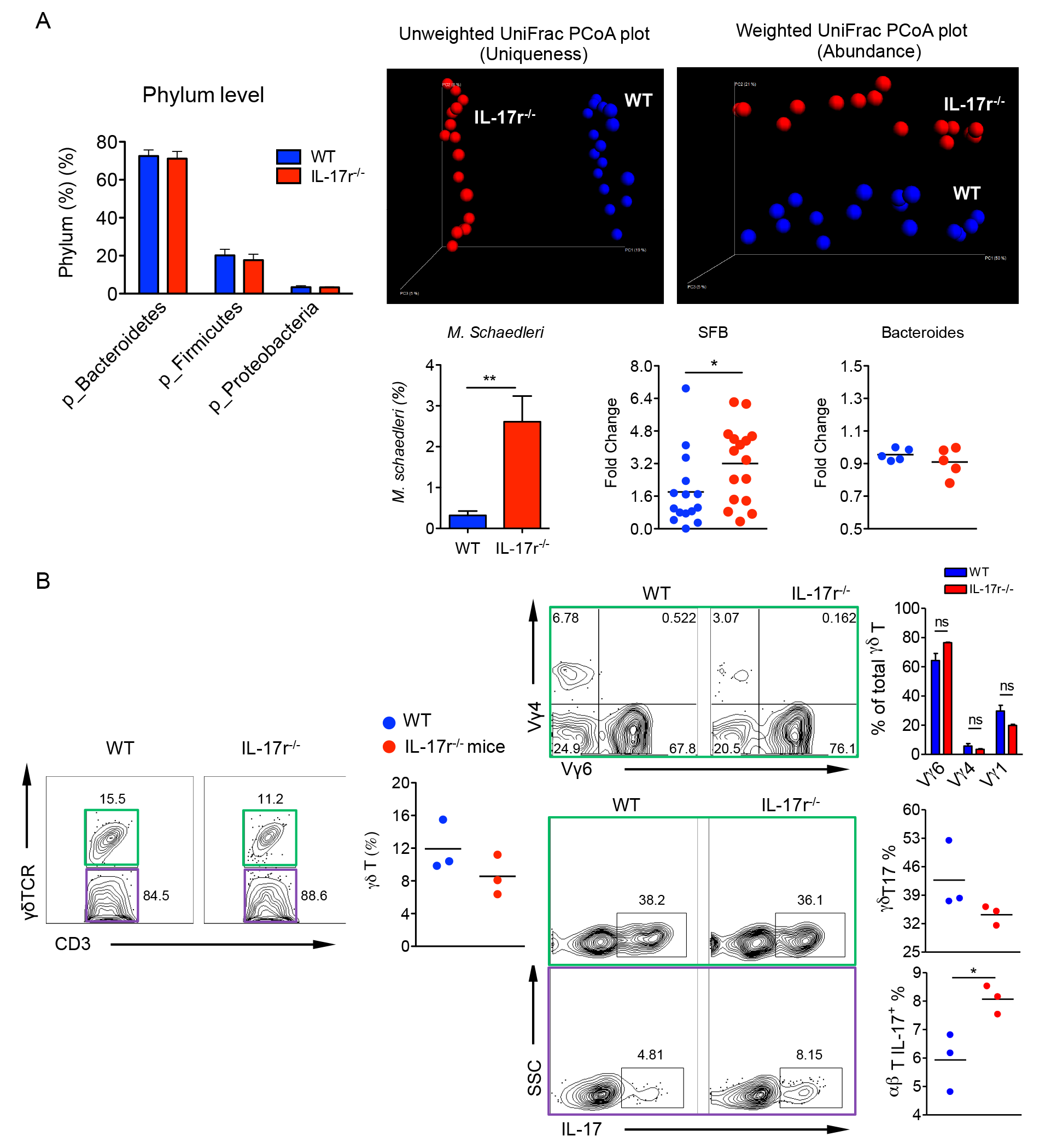


**Figure S3. Differences in gut microbiota in IL-17r^-/-^ mice does not lead to a difference in lamina propria γδ T population but does lead to increase in Th17 responses.**

1. Bacterial genomic DNA was extracted from the fecal droppings of 15 WT and 15 IL-17r^-/-^ then using deep sequencing to analyze for differences in microbiota populations. RT-PCR was used in order to confirm SFB and Bacteroides findings. *p<0.05, **p<0.01
2. Gating on total live CD45^+^ cells in the lamina propria then gated CD3 vs γδTCR to analyze γδT % and αβ T %. From these two populations were gated further looking at Vγ expression in γδ population (upper panel) as well as IL-17^+^ cells in the γδT and αβ T populations (lower panel). Five hour PMA/I stimulation was used to look at IL-17 responses. Representative of 3 experiments. *p<0.05

**Supplemental Figure 4**


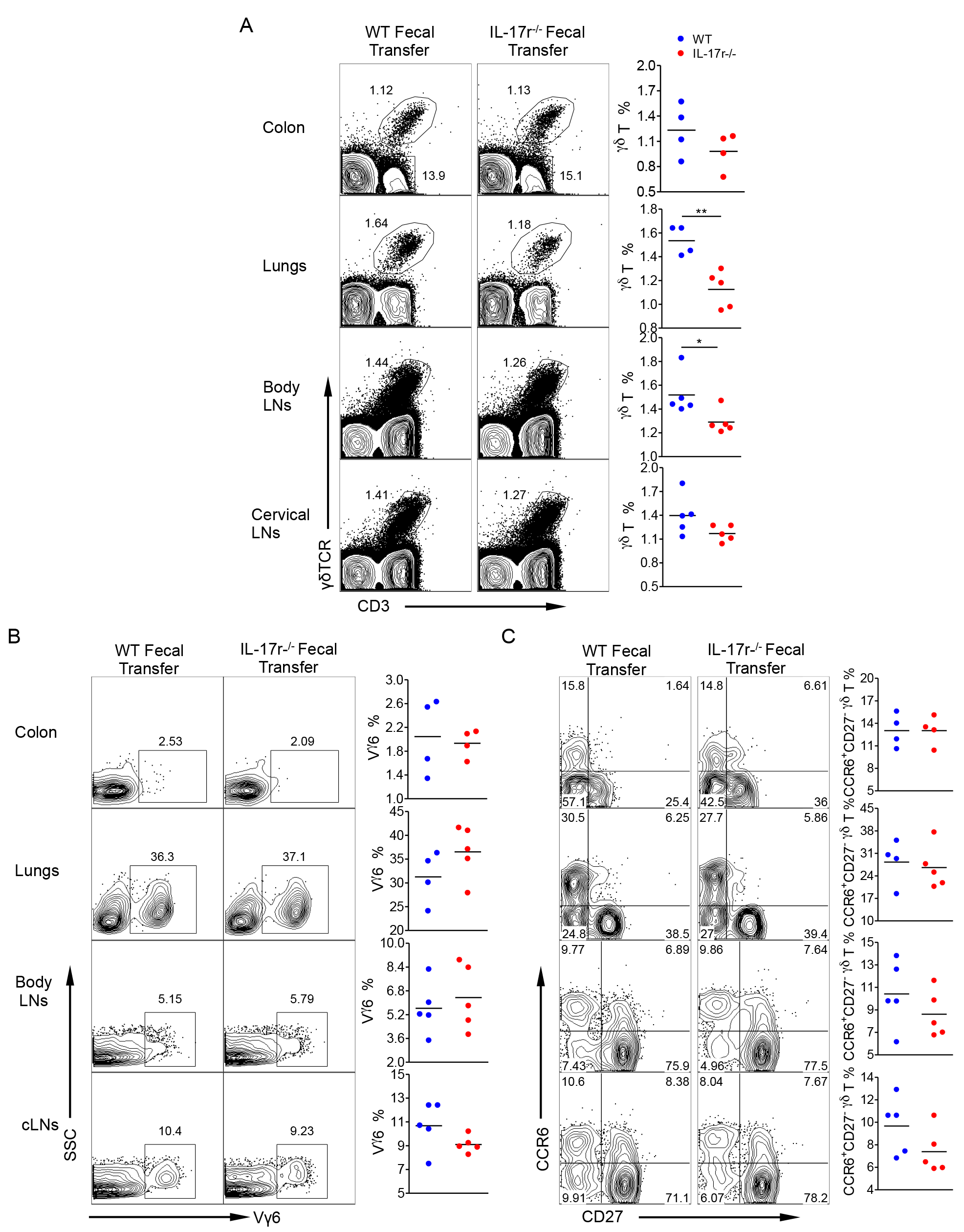


**Figure S4. Fecal transfer from IL-17r^-/-^ mice to WT mice does not induce γδT17 cell expansion**

Gastric gavage needle injection was used to do 3 times treatment of 12 mg IL-17r^-/-^ or WT fecal matter to 10 WT mice in order to determine whether IL-17r^-/-^ gut microbiota directly induces a change in γδT populations and γδT17 expansion.

1. Gating on total live lymphocyte population and then gated on CD3 vs. γδTCR to quantify γδ T %. *p<0.05, **p<0.01.
2. Gating on the total γδT population and then gated for Vγ6^+^ %.
3. Gating on total γδT cells and then gated for CD27 vs CCR6^+^ in order to analyze the CCR6^+^CD27^-^ potential γδT17 %.

**Supplemental Figure 5**


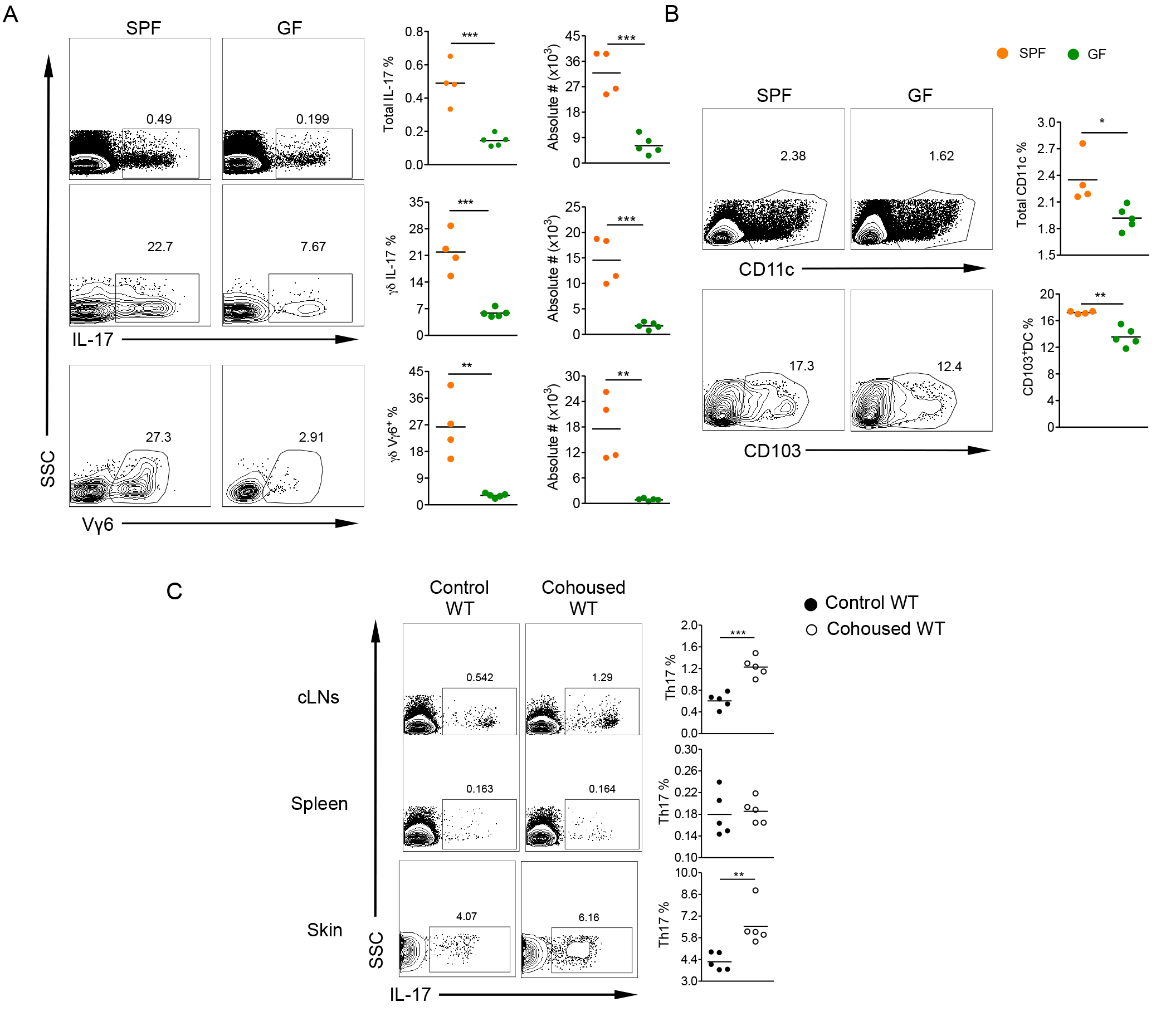


**Figure S5. Cohousing of IL-17r^-/-^ mice with WT mice induces the proliferation of Th17 cells**

Flow cytometry studies of immunostained homogenized cLNs from GF and SPF mice of similar age and sex from the same facility (U. of Chicago, Chicago, IL) **p<0.01, ***p<0.001

1. Gating from the total live cells stimulated with PMA/Ionomycin for 5 h then gated IL-17^+^ population (upper panel), gating from the total γδT cell then gated on IL-17^+^ population (middle panel) and gated on Vγ6 population (lower panel). Absolute numbers are also shown. **p<0.01, ***p<0.001
2. Gating from total live cells then gated CD11c^+^ population (upper panel) and gating from CD11c^+^ population then gated CD103^+^ population. *p<0.05, **p<0.01
3. Flow cytometry analysis of cell homogenate from different tissues looking at the intracellular expression of IL-17 from gated CD4 T cells. Gating on total live lymphocyte population then gated for CD3^+^CD4^+^ T cells to analyze Th17 responses after 5 h PMA/Ionomycin stimulation. **p<0.01, ***p<0.001
